# Promotion and consumption of commercially produced foods among children: situation analysis in an urban setting in Senegal

**DOI:** 10.1111/mcn.12304

**Published:** 2016-04-15

**Authors:** Alison B. Feeley, Aminata Ndeye Coly, Ndeye Yaga Sy Gueye, Elhadji Issakha Diop, Alissa M. Pries, Mary Champeny, Elizabeth R. Zehner, Sandra L. Huffman

**Affiliations:** ^1^ JB Consultancy Bryanston South Africa; ^2^ Helen Keller International Dakar Senegal; ^3^ Helen Keller International, Africa Regional Office Dakar Senegal; ^4^ Helen Keller International, Asia Pacific Regional Office Phnom Penh Cambodia; ^5^ Helen Keller International Washington DC USA; ^6^ Consultant to Helen Keller International

**Keywords:** complementary foods, complementary feeding, breast milk substitutes, infant feeding, infant and child nutrition, child feeding

## Abstract

This study assessed the promotion of commercially produced foods and consumption of these products by children less than 24 months of age in Dakar Department, Senegal. Interviews with 293 mothers of children attending child health clinics assessed maternal exposure to promotion and maternal recall of foods consumed by the child on the preceding day. Promotion of breastmilk substitutes and commercially produced complementary foods outside health facilities was common with 41.0% and 37.2% of mothers, respectively, reporting product promotions since the birth of their youngest child. Promotion of commercially produced snack food products was more prevalent, observed by 93.5% of mothers. While all mothers reported having breastfed their child, only 20.8% of mothers breastfed their newborn within the first hour after delivery, and 44.7% fed pre‐lacteal feeds in the first 3 days after delivery. Of children 6–23 months of age, 20.2% had consumed a breastmilk substitute; 49.1% ate a commercially produced complementary food, and 58.7% ate a commercially produced snack food product on the previous day. There is a need to stop the promotion of breastmilk substitutes, including infant formula, follow‐up formula, and growing‐up milks. More stringent regulations and enforcement could help to eliminate such promotion to the public through the media and in stores. Promotion of commercial snack foods is concerning, given the high rates of consumption of such foods by children under the age of 2 years. Efforts are needed to determine how best to reduce such promotion and encourage replacement of these products with more nutritious foods.

## Introduction

Despite an increasing focus on children's health and nutrition in the last decade in Senegal, there is room for improvement (Wuehler & Ly Wane 2011); 19% of Senegalese children less than 5 years of age are stunted, and 60% are anaemic (ANSD 2015). Breastfeeding is widely practiced, with 99% of children ever breastfed. Continued breastfeeding at the age of 1 year is also high, at 94%. However, rates of exclusive breastfeeding (which is associated with reduced morbidity and mortality) are low; just 33% of Senegalese children less than 6 months of age are exclusively breastfed. National data also indicate sub‐optimal complementary feeding practices, which can adversely impact child growth and development. Only 8% of children between 6 and 23 months of age met the minimum standard with respect to three infant and young child feeding indicators (adequate feeding frequency, minimum dietary diversity and consumption of breastmilk/other milks)(ANSD 2015).

Promotion of breastmilk substitutes (BMS), including infant formula, follow‐up formula and growing‐up/toddler milks, is potentially detrimental to child health by resulting in sub‐optimal breastfeeding behaviours (Piwoz & Huffman 2015). In response to unethical marketing activities by infant formula manufacturers and distributors, Senegal's 1994 ‘Inter‐ministerial Decree Establishing the Conditions for Marketing Breast‐milk Substitutes’ includes explicit provisions prohibiting the free distribution, promotional sale, advertising, or idealizing representations of BMS (including products, foods, or liquids used or presented as products for partial or complete replacement of breastmilk) within health facilities associated with the Ministry of Health and Social Action. The Decree does not place restrictions on the marketing of these products *outside* of these facilities in the media or retail outlets (Republic of Senegal, Ministry of Health and Social Action, Ministry of Trade and Handicrafts 1994).

Marketing of commercially produced complementary foods (CPCF), if not represented as a total or partial replacement for breastmilk, is not regulated by Senegalese legislation nor is the marketing of commercially produced snack foods. CPCFs can contribute to the improved nutritional status of children between 6 and 23 months if they are appropriately formulated (PAHO/WHO 2003) (WHO & UNICEF 2003). However, commercially produced snack foods may be detrimental to the optimal feeding of children aged 6–23 months by potentially increasing the consumption of foods high in salt, sugar, or trans‐fatty acids and by displacing the consumption of other more nutritious foods (Tzioumis *et al*. 2015). While 13 Demographic and Health Surveys (DHS) in sub‐Saharan Africa gathered information on consumption of ‘sugary snack’ foods (Huffman *et al*. 2014), this was not measured in the Senegal DHS (ANSD 2012).

Our research assessed consumption of commercially produced foods including BMS, CPCF and various snack food products consumed by children less than 2 years of age as reported by their mothers, and maternal recall of promotions of these products. This research was part of a four‐country study assessing these issues in Cambodia, Nepal, Senegal and Tanzania (2016a, 2016b, 2016c, 2016d; Vitta *et al*. 2016; Champeny *et al*. 2016; Sweet *et al.* 2016; Pereira *et al*. 2016).

**Key messages**

Our findings could be used as part of a situation analysis on IYCF in Senegal to help develop a plan to scale up improved IYCF. Specifically:

- health workers should be discouraged from recommending BMS and permitting its use for pre-lacteal feeds in health facilities;
- commercial promotion of BMS outside health facilities is common and should be prohibited;
- promotion of commercial snack foods outside health facilities is common and should be regulated; and
- mothers need information on the health consequences of feeding young children BMS and commercial snack foods and on benefits of breastmilk and locally produced healthy complementary foods.

## Materials and methods

This was a cross‐sectional survey using a multi‐stage sampling procedure to obtain a representative sample of mothers of children <24 m of age utilizing public/faith‐based/non‐governmental health facilities associated with the Ministry of Health and Social Action in urban areas of Dakar Department in Dakar Region, Senegal. Approval for this study was obtained from Senegal's National Ethics Committee for Research in Health on 10 March 2014, prior to data collection. Written informed consent was obtained from all participants prior to the interview being conducted. Interviewers read the consent form to mothers, and those who agreed to participate were asked to sign the form and were given a copy of the consent form.

Data were collected through structured interviews among mothers of children less than 24 months of age who were utilizing child health clinics. Data collection took place between April and June 2014. Mothers living outside of Dakar Department, but utilizing child health services in the Department, were excluded from participating in the survey. Additionally, because of possible impacts on breastfeeding, mothers were excluded if they experienced severe delivery complications or if the child had a congenital disease, was in the neonatal intensive care unit, was too ill for the mother to be interviewed or was from a multiple birth (as multiple births may be considered a risk factor for exclusive breastfeeding).

The sample size for this study was calculated to detect a 10% prevalence rate of exposure to promotions within the health system or to detect a 10% prevalence rate of children's consumption of commercially produced food on the preceding day with a measurement error of ±5%. Using a standard of error of 0.0255 and assuming a design effect of 2 to account for the cluster design, a sample size of 280 for mothers of children less than 24 months of age was calculated.

Lists of all public/faith‐based/non‐governmental organisation (NGO) health facilities offering child health services in Dakar were obtained from the National Information Service for Health. This included national hospitals, referral hospitals and health centres but excluded health posts. Utilization numbers for these facilities provided the number of child health visits (out‐patient departments and immunization clinics). The number of visits was then calculated as the monthly average number of visits per facility; if annual data were obtained, the number of visits was divided by 12 months. Private facilities were not included because their number of visits was low and it would have been logistically difficult to obtain sufficient numbers of child health visits during the data collection period. Additionally, the Senegal DHS (2010–2011) reported only 10.5% of health facility births in Dakar occur in private facilities (ANSD 2012) suggesting that the use of private facilities for child health visits may be similarly low.

Public/faith‐based/NGO health facilities were sampled by allocating clusters using probability proportional to size. The calculated monthly utilization numbers served as each facility's ‘population’. Because of logistics and the need to complete data collection within 3 months, facilities with less than 50 child health visits per month were excluded from the sampling frame. This excluded 26 out of 81 child health facilities, but the 55 included in the sampling frame represented 97.6% of all child health visits in Dakar Department public/faith‐based/NGO health facilities.

Eighteen clusters were sampled among facilities with at least 16 mothers each in each cluster to allow for even distribution of child ages across four age categories (0–5.9, 6–11.9, 12–17.9, and 18–23.9 months). In a limited number of facilities, more than 16 mothers were recruited to fulfill necessary age ranges because of the recalculation of ages by spss subsequent to interviews that resulted in slight changes in the age of the child compared with calculations made in the field.

**Figure 1:**
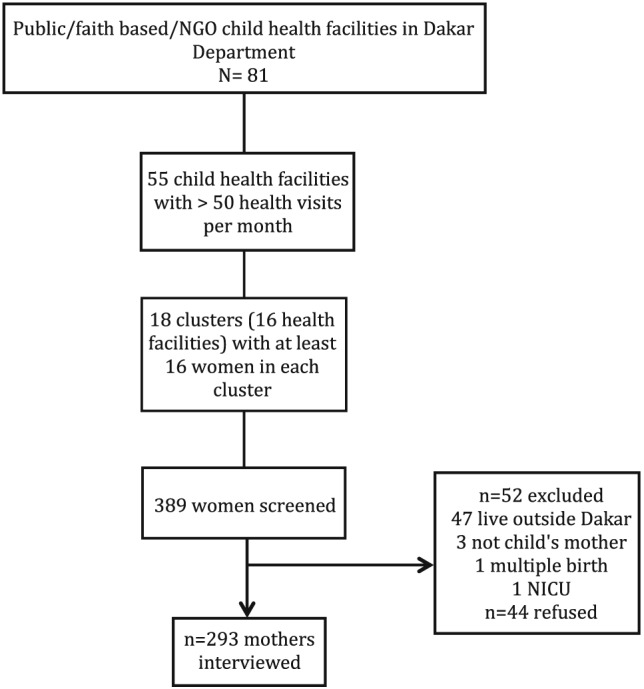
Sampling of health facilities and mothers.

Because sampling of facilities was proportional to size, larger facilities had a greater chance of being sampled for multiple clusters compared with smaller facilities. Figure 1 details the sampling of facilities and mothers in the study.

Sampled facilities were alerted about data collection approximately 1 week prior to the survey. Women with children visiting sampled health facilities were approached for interview by survey supervisors who screened every woman with a child who passed through the entrance/exit point of the child health clinic area and assessed the age of the child to verify if an interview was still needed for the specific age category.

Prior to being assigned to an enumerator, all mothers in child out‐patient/immunization services were first screened against the exclusion criteria by survey supervisors in order to save time for both mothers and enumerators. A total of 389 mothers with children under 24 months of age were approached for interview. Of these, 52 (13.4%) mothers were excluded from the study based on one or more of the exclusion criteria: 47 mothers resided outside of Dakar, and three women interviewed were not the mother of the child; one mother's youngest child was from a multiple birth, and one child had been in the neonatal intensive care unit after delivery. Forty‐four (13.1%) mothers out of all eligible mothers refused participation in the study. The final sample of mothers included for interview in the study was 293.

Data to assess infant and young child feeding practices were gathered in accordance with the World Health Organization guidelines on infant and young child feeding (IYCF) practices (WHO 2008). Standardized questionnaires were used to obtain information on which foods and beverages were consumed on the day and night prior to the day of interview. Additionally, data were gathered on the weekly frequency of consumption, reasons for feeding and expenditure on commercially produced snacks to children under 2 years of age, as well as the types of foods mothers aspired to feed their youngest child and associated reasons.

Mothers were asked to report on promotional practices experienced inside and outside the health facilities for BMS, CPCF and commercially produced snack foods. In this study, BMS includes infant formula (generally for use from birth up to 6 months of age), follow‐up formula (for use from 6 months through 11 months) and other milk or milk‐like products marketed or otherwise represented as suitable for feeding children younger than 2 years of age, including growing‐up milk and toddler milks (generally recommended for use from 12 months of age). CPCF includes commercially produced food or beverage products, excluding BMS, that contain a label indicating that the product is intended for children younger than 2 years of age. CPCF includes infant cereal/porridge, pureed food, baby snacks/finger food and tea/water/juice. Mothers were asked if they had seen promotions for commercially produced snack foods including soft drinks, juice/juice drinks, savory snacks (chips, salted biscuits) and sweet snacks (sweet biscuits/cookies, candy/sweets/chocolate and cakes/doughnuts). Women who gave these foods were asked the reason why they bought these foods in the last week, and those who made home‐prepared complementary foods were asked why they did so in the preceding day.

“Exclusive breastfeeding” is defined as no other food or drink fed to the infant except breastmilk, except that the infant may have received oral rehydration solution (ORS), drops and syrups (vitamins, minerals and medicines) (WHO & UNICEF 2003). Pre‐lacteal feeding is defined as the provision of liquids other than breastmilk in the first 3 days after delivery. Minimum dietary diversity for 6–23‐month‐old children is defined as the child consuming at least four of seven food categories in the previous day. Minimum meal frequency is defined as the child consuming food the minimum number of times or more in the previous day (with the minimum depending on their age and breastfeeding status). A minimum acceptable diet is the combination of these two indicators (WHO 2008).

The questionnaires were designed in Microsoft Word and then entered in Formhub, an open‐source online platform that allows data to be collected via mobile phones or tablets, using the Android application Open Data Kit (ODK) Collect (Formhub.org 2013). The questionnaires were translated from English into French and Wolof, back translated into English to ensure accuracy and uploaded into Formhub in French and Wolof. While French is the official language in Senegal, Wolof is widely used. Wolof translations were transcribed phonetically for both the questionnaire and mothers' responses. Interviews were conducted in French or Wolof, depending on the mother's preference. Data were collected using mobile devices – Samsung Galaxy tab 2.0 7 model tablet – in order to allow for immediate data entry, reduction in data entry errors and prompt analyses. Data were submitted online directly to Formhub, and submitted questionnaires were reviewed daily to ensure data quality.

Data were cleaned and analysed using spss version 22 (IBM, Armonk, NY, USA). Proportions for categorical variables and means ± standard deviations for continuous variables were used to describe the results. Differences in predictive variables (such as child age categories) by outcome variables (such use of BMS) were assessed through bivariate comparisons using Pearson chi‐square or Fisher exact test (for 2 × 2 tables) to test for significance.

## Results

Demographic characteristics of the study respondents are shown in Table 1. About one fourth of mothers reported working outside the home. Television ownership was nearly universal, and two‐thirds of mothers reported having a refrigerator in their home.

**Table 1:** Demographic and socio‐economic characteristics of mothers and their children under 24 months of age

	Mothers with children <24 months (*n* = 293)
Mother	—
Age (years) (mean ± SD)	28.8 ± 5.7
<20 years	2.7
Parity (*n* = 287) (%)	—
Primiparous	27.9
2–3	49.5
4+	22.6
Marital status (%)	—
Married	95.9
Divorced, widowed or separated	4.1
Level of education (%)	—
None	23.5
Non‐formal education	4.1
Primary	33.1
Lower secondary	16.4
Upper secondary	10.6
Tertiary	12.3
Household	—
Safe source of drinking water (%)	99.7
Assets, ownership (%)	—
Refrigerator	68.9
Television	95.9
Antenatal and delivery care	—
Received antenatal care (%)	98.9
Assisted at delivery by (%)	—
Physician	19.5
Midwife	78.2
Caesarian delivery (%)	13.2%
Child characteristics	—
Age (%)	—
0–5 months	25.6
6–11 months	24.6
12–17 months	25.3
18–23 months	24.6
Sex (female) (%)	51.0

SD, standard deviation.

### Promotion of breastmilk substitute, commercially produced complementary food and commercial snack food products

One‐fifth (21.2%) of mothers reported that since the birth of their child, health workers had recommended that they use BMS, with no difference by the child's age (*P* = 0.407). Only 6.8% of mothers reported that health workers recommended CPCF, with recommendations greater for older children: 1.3% and 1.4% of those 0–5 months and 6–11 months compared with 8.1% and 16.7% of those with children 12–17 months and 18–23 months (*P* = 0.001). Mothers' reported exposure within health facilities to advertisements for BMS and CPCF was low, with only 2.0% of mothers reported observing ads for either BMS or CPCF. However, more (19.8%) observed branding for a BMS or CPCF product on health facility equipment or materials (mostly on posters) within the health facility (Table 2).

**Table 2:** Percentage of mothers who reported having heard, seen or read a promotion for BMS or CPCF by type of promotion (*n* = 293)

Type of promotion	Any IYCF product	Breastmilk substitutes	Commercially produced complementary foods
Promotions in health facilities	—	2.0	2.0
Branding on health facility equipment	19.8	NA*	NA*
Posters	16.0	—	—
Pads of paper	2.7	—	—
Decorations	2.4	—	—
Blankets	1.0	—	—
Promotions outside health facilities	—	41.0	37.2
Where observed promotion	—	—	—
Television	—	38.9	34.1
Pharmacies	—	6.8	5.5
Billboards	—	4.4	4.4
Radio	—	1.0	3.8
Internet	—	1.4	1.7
Types of products promoted	—	—	—
BMS	—	—	—
Infant formula	—	16.7	—
Follow‐up formula	—	17.1	—
Growing‐up milk	—	14.0	—
CPCF	—	—	—
Infant cereal	—	—	29.4

* Respondents not asked to report by type of product.

BMS, breastmilk substitute; CPCF, commercially produced complementary food; IYCF, infant and young child feeding.

**Figure 2:**
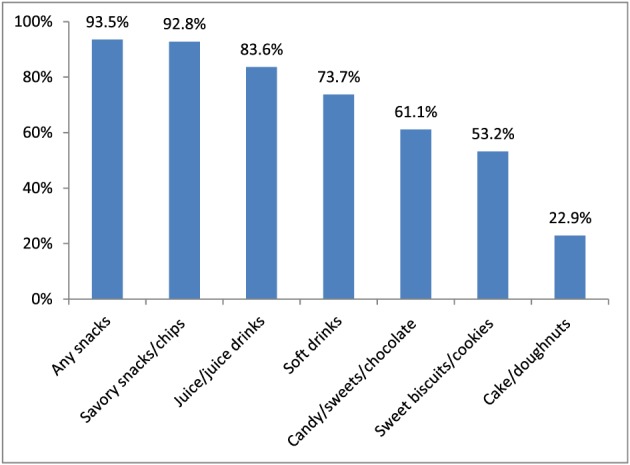
Percentage of mothers who reported having heard, seen or read a promotion of commercially produced snack foods and soft drinks by type of product (*n* = 293).

About 40% of mothers saw, heard or read a promotion outside health facilities for BMS or CPCF (Table 2). Most of these promotions were advertisements on television. Mothers reported observing promotions for all three types of breastmilk substitutes (infant formula, follow‐up formula and growing‐up milks). The most common product reported in CPCF promotions was infant cereal. Promotions for any commercially produced snack food products and soft drinks were reported by nearly all mothers (Fig. 2). Commercially produced savory snacks/chips were the most commonly reported product category promoted.

### Child feeding practices

All children less than 24 months of age had ever been breastfed, but optimal breastfeeding practices were low: just one‐fifth of mothers breastfed within the first hour after delivery, and nearly one‐fifth of infants were given BMS in the first 3 days after delivery, with infants born to mothers with caesarian deliveries more likely to have been given pre‐lacteal BMS than those with mothers who had vaginal deliveries (Table 3). Only one‐third of infants less than 6 months of age were exclusively breastfed. While only one child (out of 52 infants) aged 0–3.9 months consumed semi‐solid or solid foods on the preceding day, 39.1% of 23 infants 4.0–5.9 months of age did so (*P* = 0.000).

**Table 3:** Percentage of mothers who reported that they breastfed within the first hour after delivery, whose infants were fed BMS as a pre‐lacteal feed, and who exclusively breastfed by type of delivery

	Vaginal delivery		Caesarian delivery		Total		*P* value
	*n*	%	*n*	%	*n*	%	
Breastfed within first hour after delivery	240	23.2	38	13.2	278	21.9	0.112
Infants fed BMS as pre‐lacteal feed	253	14.2	40	45.0	293	18.3	0.000
Exclusively breastfed (among infants < 6 months of age)	63	36.5	12	25.0	75	34.7	0.339

BMS, breastmilk substitute.

Table 4 shows the reported consumption of different liquids and semi‐solid/solid foods by children under 2 years of age on the previous day. Minimum dietary diversity was met by 48.6%; minimum meal frequency was met by 50.5%, and a minimum acceptable diet was achieved by one‐third (32.1%) of children 6–23 months of age. Nearly 20% (18.2%) of all children were fed with a bottle on the preceding day, with no difference by age of the child (*P* = 0.592).

**Table 4:** Percentage of children reported to have consumed specified beverages and foods on the preceding day by age of child

Type of food	<6 months of age (*n* = 75)	6–23 months of age (*n* = 218)	*P* value	6–11 months of age (*n* = 72)	12–17 months of age (*n* = 74)	18–23 months of age (*n* = 72)	*P* value
**Liquids**	—	—	—	—	—	—	—
Breastmilk	100.0	76.6	0.000	95.8	91.9	41.7	0.000
Plain water	6.7	73.9	0.000	55.6	78.4	87.5	0.000
Tinned/powdered milk	2.7	35.3	0.000	11.1	36.5	58.3	0.000
Juice/juice drink	0.0	30.7	0.000	11.1	39.2	41.7	0.000
Purchased juice	0.0 (n=0)	77.6 (n=67)	0.000	37.5 (*n* = 8)	79.3 (*n* = 29)	86.7 (*n* = 30)	0.012
Bottled water	48.0	30.3	0.005	43.1	28.4	19.4	0.008
Tea/infusions/coffee	0.0	22.5	0.000	6.9	24.3	36.1	0.001
Breastmilk substitute (BMS)	10.7	20.2	0.042	22.2	18.9	19.4	0.868
Soft drinks	0.0	7.8	0.006	4.2	9.5	9.7	0.372
Sugar/honey added to liquids	9.3	11.9	0.692	11.1	9.5	15.3	0.534
**Semi‐solid/solid foods**	—	—	—	—	—	—	—
Cereal‐based foods	10.7	86.7	0.000	75.0	91.9	93.1	0.002
Yogurt	9.3	63.3	0.000	62.5	66.2	61.1	0.803
Butter, oil or fat	12.0	60.1	0.000	47.2	66.2	66.7	0.024
Sugar or honey added to food	9.3	60.1	0.000	50.0	64.9	65.3	0.102
Yellow/orange flesh vegetables	1.3	52.3	0.006	47.2	62.2	47.2	0.112
Savory snacks	1.3	49.1	0.000	23.6	59.5	63.9	0.000
Sugary snacks	0.0	47.7	0.000	18.1	58.1	66.7	0.000
Potatoes	2.7	43.1	0.000	33.3	52.7	43.1	0.061
Fish or seafood	0.0	36.7	0.000	15.3	43.2	51.4	0.000
Other fruits and vegetables	1.0	27.5	0.000	19.4	27.0	36.1	0.081
Cheese	2.7	25.7	0.000	16.7	31.1	29.2	0.098
Eggs	0.0	20.6	0.000	5.6	29.7	26.4	0.001
Mango/papaya	0.0	11.5	0.000	1.4	12.2	20.8	0.001
Dark green leafy vegetables	0.0	10.1	0.000	5.6	12.2	12.5	0.295
Beans or lentils	0.0	8.7	0.003	1,4	8.1	16.7	0.005
Peanut butter	0.0	8.7	0.003	2.8	8.1	15.3	0.028
Meat or poultry	0.0	7.8	0.006	1.4	9.5	12.5	0.037
Nuts	0.0	6.0	0.019	0.0	5.4	12.5	0.006
**Commercially produced complementary foods**	8.0	49.1	0.000	43.1	54.1	50.0	0.406
Infant cereal	6.7	26.1	0.000	27.8	31.1	19.4	0.258
Puree	2.7	11.9	0.011	15.3	8.1	12.5	0.403
Infant snacks	0.0	27.5	0.000	5.6	28.4	33.3	0.000
**Commercially produced snack food products**	1.3	58.7	0.000	26.4	71.6	84.7	0.000
Savory snacks/chips	1.3	48.2	0.000	22.2	58.1	63.9	0.000
Sweet biscuits/cookies	0.0	24.8	0.000	5.6	32.4	36.1	0.000
Candy/sweets/chocolate	0.0	25.7	0.000	6.9	29.7	40.3	0.000
Cake/doughnuts	0.0	8.8	0.410	3.3	9.0	13.4	0.132
Soft drinks	0.0	7.8	0.006	4.2	9.5	9.7	0.372

Of children 6–23 months of age, half (50.5%) had consumed a homemade complementary food, which was defined as a food that a household member prepared at home that is especially for babies. Similarly, half (49.1%) had consumed a CPCF, with about one‐fourth having consumed an infant cereal and one‐fourth an infant snack.

The most common foods eaten on the preceding day by children 6–23 months of age included breastmilk, cereal‐based foods (e.g. rice, wheat and millet), yogurt, butter, oil or other fat, sugar or honey added to food and commercial snack foods/soft drinks. Chips were the most commonly consumed commercial snack food, with nearly two‐third of children 18–23 months having eaten them on the preceding day.

**Figure 3:**
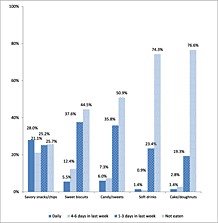
Percentage of mothers reporting that their children 6–23 months of age consumed commercially produced snack food products or soft drinks in the last week by frequency of consumption (*n* = 218).

**Figure 4:**
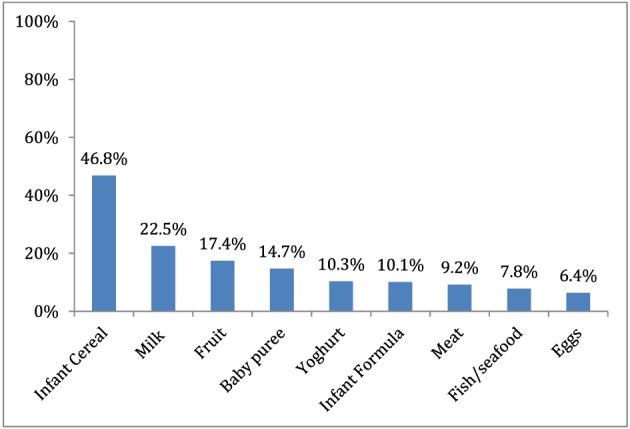
Percentage of mothers of children 6–23 months of age who reported they would feed their children other foods if they could afford them by foods mentioned (*n* = 204).

**Figure 5:**
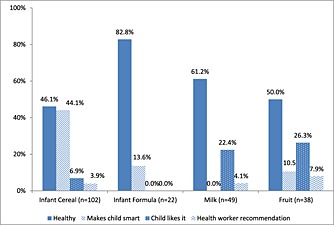
Percentage of mothers of children 6–23 months of age reporting reasons for wanting to buy and feed additional foods to their child if they could afford them by type of food.

Of children aged 6–23 months, 79.8% had consumed a commercially produced snack food in the previous week. The percentage who consumed one, two, three or four snack foods on the preceding day was 27.1%, 18.8%, 9.6% and 3.2%, respectively. About half had eaten them daily or nearly every day (Fig. 3). When asked why they fed homemade complementary foods, commercial infant cereal or commercially produced snack food to their child in the last week, the most commonly reported reason by mothers was because the ‘child likes it’ (Table 5). While less than half of mothers reported this as the reason for feeding CPCFs or home‐prepared complementary foods, over 80% of mothers reported this as the reason for feeding a commercial snack food. Three‐fourths of mothers reported they would feed their child additional foods if they could afford them. Commercial infant cereal was the most commonly reported food (Fig. 4) with nearly half stating that the reason they wanted to offer this food was because it “makes baby smart” (Fig. 5). Of mothers who reported wanting to feed infant formula to their child, the most commonly reported reason was because it was healthy.

**Table 5:** Percentage of mothers who gave reasons for feeding/buying select foods for children 6‐23 mo of age

Type of food (*n* = mothers reporting reasons for giving/buying this food)	Reason given (%)									
	Child likes it	Convenience	Healthy	To vary diet	Ads say they are good for child	To calm child	Affordable	Traditionally done	Other	Total
Homemade complementary food (*n* = 110)	37.3	21.8	19.1	6.4	0.9	—	0.9	1.8	11.8	100.0
Commercial infant cereal (*n* = 57)	45.6	17.5	12.3	—	5.3	—	1.8	1.8	15.8	100.1
Commercially produced snack foods	—	—	—	—	—	—	—	—	—	—
Savory snacks/chips (*n* = 162)	88.3	1.9	1.2	—	—	—	3.1	0.6	4.9	100.0
Sweet biscuits/cookies (*n* = 121)	80.2	4.1	0.8	—	—	4.1	1.7	0.8	8.3	100.0
Sweets/candy (*n* = 107)	86.0	—	0.9	—	—	1.4	4.7	—	7.0	100.0
Cakes (*n* = 51)	86.3	5.9	—	—	—	—	—	—	7.8	100.0
Soft drinks (*n* = 56)	75.0	1.8	—	—	—	8.9	—	—	14.3	100.0

Mothers who purchased commercially produced snack food products or soft drinks in the last week reported spending on average per day 54.28 West African CFA francs ($US.10) on sweet biscuits/cookies, 17.14 West African CFA franc ($US.003) on sweets/candy/chocolate, 50.02 West African CFA franc ($US.09) on chips/savory snacks, 95.38 West African CFA franc ($US.18) on cakes/doughnuts and 94.41 Francs CFA ($US.18) on soft drinks. 1
http://www.xe.com/currency/xof-cfa-franc, 24 October 2014, $US1 = 518.050 CFA Franc.


## Discussion

Commercial promotion of BMS and commercial snack foods are likely to be one of the contributing factors for poor feeding practices observed in Dakar Department, Senegal. The use of BMS in the first 3 days after delivery was common, and exclusive breastfeeding rates were low in this study. Although promotion of BMS in health facilities is prohibited by the International Code of Marketing of Breast‐milk Substitutes (WHO 1981) and the Inter‐ministerial Decree in Senegal, our findings indicate that many mothers received recommendations from health workers to use BMS and observed infant feeding brands on health facility equipment. Commercial promotion for BMS outside health facilities was frequently observed with nearly 40% of mothers having seen advertisements on television. Mothers reported seeing advertisements for all three types of products: infant formula, follow‐up formula and growing‐up milks. Such products are often cross‐promoted with similar branding, names and packaging. Among mothers who reported that they would feed BMS to their children if they could afford it, the reason given by the majority of these mothers was because it was considered healthy for the child. In a review of 36 BMS labels of products sold in Senegal in 2014, all included wording suggesting nutritional or health benefits of the products (Sy Gueye 2015).

Nearly two‐thirds of children 6–23 months of age consumed commercially produced snack foods/soft drinks. Only breastmilk, foods made from cereals and added sugar/honey or fats were as frequently consumed on the preceding day as commercial snack products. Chips were the most commonly consumed snack, with 3/4ths of children eating them in the last week. Given that a breastfed child from 6–23 months of age needs from just 250 to 550 additional calories per day depending on age (Dewey & Brown 2003), the consumption of such typically highly caloric snack foods may be a reason why only one‐third of these urban children had a minimally acceptable diet.

Strikingly, twice as many mothers reported seeing promotions of commercially produced snack food products and soft drinks as seen in CPCF promotions. The major reason why mothers reported giving these snack foods was because the child “liked them”. This may also explain why nearly two‐thirds of mothers reported adding sugar or honey to either a food or beverage fed to their child. Such feeding practices are the cause for concern given that consumption of energy‐dense, nutrient‐poor foods that are high in sugar or salt can contribute to child overweight and obesity and/or in the long term, elevated risks of non‐communicable diseases (Lustig *et al*. 2015; WHO & UNICEF 2003). Additionally, funds spent for snack foods can result in less being available for foods with higher nutrient content. Mothers reported that they spent around $.10 per day to purchase chips and sweet biscuits, a substantial amount given that a locally produced fortified complementary food costs $.13 and an imported one cost $.45 per serving in Dakar (Pereira *et al*. 2014).

Other research suggests that commercial snack foods are packaged in a way that is appealing to young children and are often inexpensive (Sweet *et al*. 2016), which may contribute to their popularity. However, the fact that nearly half of mothers reported that they would feed their children with infant cereal if they could afford it because these products will “make the child smart” may indicate that mothers in Dakar do aspire to use CPCF, although their higher cost (compared with commercially produced snack foods) is prohibitive. In a recent survey of television advertisements for infant and young child feeding products in Dakar, 69 CPCF advertisements were observed over a 3 month period. Advertisements making reference to intelligence were observed in 81% of these adverts. This reference appeared in one brand's advertisement, which used the promotional message “cereal for the little smart ones”, and this advertisement aired 56 times in the 3‐month period. (Sy Gueye *et al*. 2016).

By assessing practices in health facilities rather than in the community, the study was able to be conducted within a few months; however, this limits the generalizability of results. The exclusion of private facilities could have led to lower percentages of wealthier mothers in our study than in the Dakar population, and promotion practices may differ between public and private facilities. Had private facilities been included, this may have led to even higher rates of BMS and commercial snack food use being reported. Another limitation of the study is the lack of quantifiable results on promotion, because we asked if a mother had observed any promotions since the birth of her child, but not how many. Additionally, mothers of older children were more likely to have the opportunity to observe a promotion.

Less than one‐fifth of countries worldwide have adopted most of the International Code provisions into national law (IBFAN‐ICDC 2011). In Africa, only 16 out of 47 countries reported fully prohibiting sales promotion of BMS (WHO 2013). This is a rate similar to that seen worldwide of 34.7% even though the Code was passed over 30  years ago by the World Health Assembly in 1981. While several African countries have updated their laws implementing the Code (including Cameroon, Nigeria and Zambia), Senegal is one of many African countries that operate under less stringent national regulations or none at all. While Senegal's *Inter‐ministerial Decree Establishing the Conditions for Marketing Breast‐milk Substitutes* restricts promotion *inside* Ministry of Health facilities, it does not apply to promotion *outside* of health facilities. Furthermore, neither Senegal nor any other country in Africa bans the promotion of commercial snack foods to the general public, although some restrict marketing to young children.

An inspection of Senegalese health facilities is warranted to remove prohibited promotional items and to investigate health workers' knowledge of the Code and Senegalese legislation and perceptions as to what constitutes a violation. This could help prevent health workers recommending BMS to mothers and allowing BMS companies to promote products in health facilities. Additionally, efforts are needed to reverse the high use BMS in health facilities by nearly half of newborns whose mothers had caesarian deliveries.

The findings from this study serve as a reminder to the international infant and young child feeding community that even in a country where improvements to infant and young child nutrition have been made, monitoring and enforcement of the Code and relevant country legislation is necessary. Marketing of snack foods is an increasing issue that should not be overlooked. These study findings also highlight a number of important issues that need to be addressed to improve young child feeding practices in Senegal and many other African countries. Our findings could be used as part of a situation analysis on IYCF in Senegal to help develop a plan to scale up improved IYCF. Specifically,

1. health workers should be discouraged from recommending BMS and permitting its use for pre‐lacteal feeds in health facilities;
2. commercial promotion of BMS outside health facilities is common and should be prohibited;
3. promotion of commercial snack foods outside health facilities is common and should be regulated; and
4. mothers need information on the health consequences of feeding young children BMS and commercial snack foods and on benefits of breastmilk and locally produced healthy complementary foods.

## Source of funding

Funding for this research was provided by the Bill & Melinda Gates Foundation.

## Conflicts of interest

The authors have no conflicts of interest to declare.

## Contributions

All authors reviewed and provided input on the final article. AF analyzed the data and drafted the manuscript. AC and NSG managed data collection and translation. EZ, SH, and AP conceptualized and designed the study, with input from ED. AP oversaw enumerator training and data collection. MC oversaw questionnaire development and technology for data collection. SH revised the final manuscript.
